# An Egg-Derived Sulfated *N*-Acetyllactosamine Glycan Is an Antigenic Decoy of Influenza Virus Vaccines

**DOI:** 10.1128/mBio.00838-21

**Published:** 2021-06-15

**Authors:** Jenna J. Guthmiller, Henry A. Utset, Carole Henry, Lei Li, Nai-Ying Zheng, Weina Sun, Marcos Costa Vieira, Seth Zost, Min Huang, Scott E. Hensley, Sarah Cobey, Peter Palese, Patrick C. Wilson

**Affiliations:** a Department of Medicine, Section of Rheumatology, University of Chicago, Chicago, Illinois, USA; b Department of Microbiology, Icahn School of Medicine at Mount Sinai, New York, New York, USA; c Department of Ecology and Evolution, University of Chicago, Chicago, Illinois, USA; d Department of Microbiology, Perelman School of Medicine, University of Pennsylvania, Philadelphia, Pennsylvania, USA; St. Jude Children’s Research Hospital

**Keywords:** LacNAc, anti-glycan antibodies, antibody repertoire, influenza vaccines, vaccine platform

## Abstract

Influenza viruses grown in eggs for the purposes of vaccine generation often acquire mutations during egg adaptation or possess different glycosylation patterns than viruses circulating among humans. Here, we report that seasonal influenza virus vaccines possess an egg-derived glycan that is an antigenic decoy, with egg-binding MAbs reacting with a sulfated *N*-acetyllactosamine (LacNAc). Half of subjects that received an egg-grown vaccine mounted an antibody response against this egg-derived antigen. Egg-binding monoclonal antibodies specifically bind viruses grown in eggs, but not viruses grown in other chicken-derived cells, suggesting that only egg-grown vaccines can induce antiegg antibodies. Notably, antibodies against the egg antigen utilized a restricted antibody repertoire and possessed features of natural antibodies, as most antibodies were IgM and had a simple heavy-chain complementarity-determining region 3. By analyzing a public data set of influenza virus vaccine-induced plasmablasts, we discovered egg-binding public clonotypes that were shared across studies. Together, this study shows that egg-grown vaccines can induce antibodies against an egg-associated glycan, which may divert the host immune response away from protective epitopes.

## INTRODUCTION

Influenza viruses have historically been grown in embryonated chicken eggs as a way to culture large quantities of virus, and as a result, most influenza virus vaccines are still generated using viruses grown in eggs. However, this process has the potential to change the immunogenicity of the virus, as the viruses may mutate their major surface glycoproteins hemagglutinin (HA) and neuraminidase (NA) to increase infectivity in eggs (1–5). Moreover, influenza viruses grown in eggs are often less immunogenic than viruses grown in mammalian cells (6–8) and have been shown to be less effective than mammalian cell-based influenza vaccines and recombinantly expressed HA vaccines (9, 10). Due to the inherent difference in avian versus mammalian glycosylation patterns, egg-grown vaccines may lack certain glycans that would be expressed on influenza viruses transmitted among humans. Notably, vaccine effectiveness against recent H3N2 viruses may be reduced due to the lack of a glycan on HA of H3N2 viruses grown in eggs (1).

However, slight differences in viral sequences and glycosylation patterns of HA do not fully explain why vaccine effectiveness is low, as serum from vaccinated subjects can have similar antibody titers against egg-adapted strains and viruses circulating in the population (11). Poor immunogenicity against HA may explain reductions in vaccine effectiveness, rather than egg-adapted mutations (11). It is possible that egg-grown vaccines are preferentially inducing antibodies against nonprotective viral antigens, therefore reducing vaccine effectiveness and seroconversion against protective epitopes on the HA head domain. Similar to the findings for subjects receiving egg-grown vaccines (12, 13), HA-reactive antibodies induced by vaccines grown in mammalian cells and insect cells largely induced antibodies mostly targeting the head domain of HA (14). However, whether different vaccine platforms induced antibodies against distinct influenza virus antigens other than HA is not known. As internal antigens, such as the nucleoprotein (NP), were shown to provide limited protection against infection (12), it remains to be determined how different vaccine formulations drive antibodies against distinct protective and nonprotective antigens.

To address whether these egg-grown influenza virus vaccines induced antibodies against potentially nonprotective antigens, we cloned monoclonal antibodies (MAbs) from plasmablasts (PBs), a transient antibody-secreting cell population, isolated from subjects following vaccination with egg-grown influenza virus vaccines. We show that 50% of subjects generated a PB response against an egg-derived antigen present in the vaccine. Subjects that mounted a response against the egg-associated antigen seroconverted against HA to levels similar to those in subjects that did not mount an antiegg response, indicating that egg-grown vaccines did not reduce overall secreted antibody responses against HA. We determined that the egg-derived antigen was a sulfated *N*-acetyllactosamine (LacNAc) glycan and was only present in viruses grown in the allantois of eggs, and not in viruses grown in a chicken embryo cell line or primary chicken fibroblasts. Antibodies binding the egg-derived glycan utilized a restricted repertoire and resembled natural antibodies, as antibodies were largely IgM and had a short heavy-chain complementarity-determining region 3 (H-CDR3). Moreover, we determined that egg-binding antibodies identified in our study were public clonotypes, indicating that the same antibodies were found across individual subjects that had been vaccinated with an egg-grown vaccine. Altogether, our study shows that egg-grown vaccines can induce antibodies against an egg-related glycan and that these glycan-binding MAbs resemble those produced by innate-like B cells.

## RESULTS

### Influenza virus vaccination induces antibodies against an egg-associated antigen.

**FIG 1:**
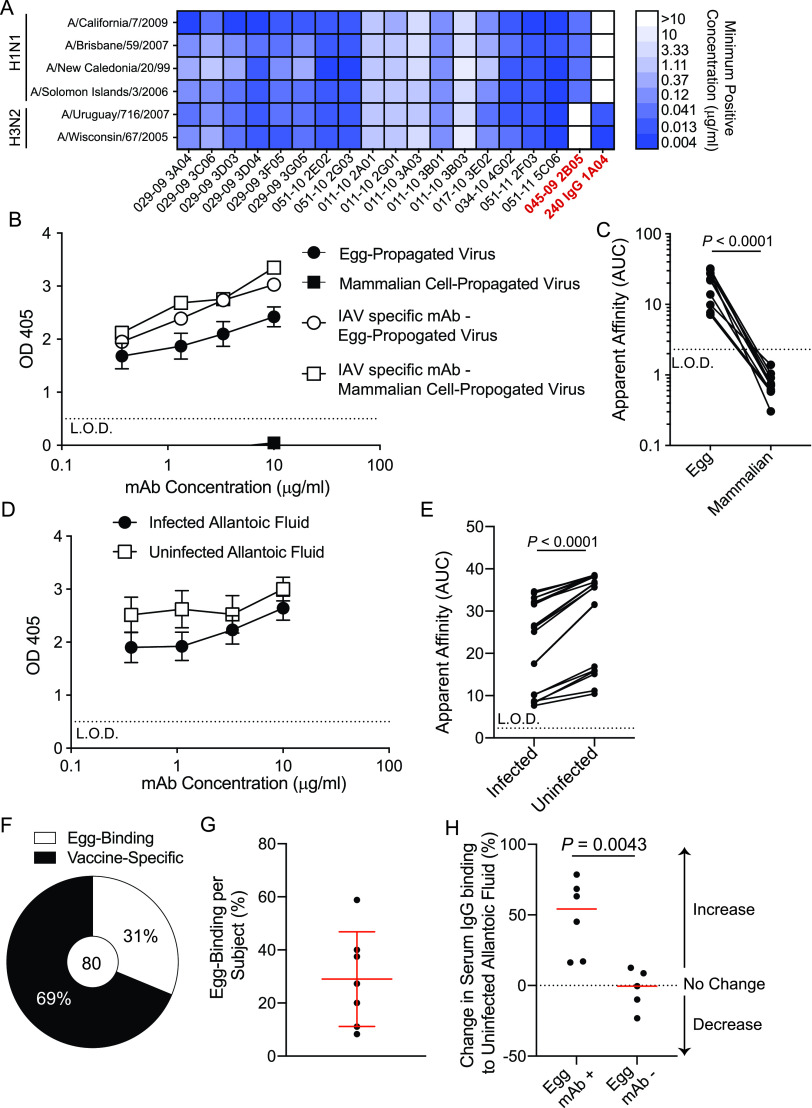
Identification of MAbs binding an egg-specific antigen. (A) Heatmap of selected MAbs binding all influenza virus strains tested. Red-highlighted MAbs are H1 specific (045-09 2B05) or H3 specific (240 IgG 1A04). Data are representative of all 27 antibodies identified. (B and C) Broadly reactive MAb (*n* = 22) binding to egg-propagated and MDCK cell (mammalian)-propagated A/California/7/2009 H1N1 (B) and apparent affinities, as calculated as area under the curve (AUC), of MAb binding (*n* = 11 MAbs) (C). (D and E) Broadly reactive MAb binding to A/California/7/2009 H1N1-infected allantoic fluid and uninfected allantoic fluid (D) and AUCs of MAb binding (*n* = 21) (E). (F) Proportions of MAbs from subjects with egg MAbs that are egg binding or are specific to the vaccine. (G) Proportions of total MAbs that are egg binding per subject. (H) Serum was isolated from subjects with or without isolated egg-binding MAbs before and 14 to 21 days after vaccination. Relative changes in serum IgG binding to uninfected allantoic fluid are represented as percentages. Red lines represent the medians. (B, D, and G) Data are mean values ± SD. (C and E) Data were analyzed using a two-tailed Wilcoxon matched-pairs signed-rank test. (B to E) L.O.D., limit of detection. (H) Data were analyzed using a two-tailed Mann-Whitney test.

To address the antigen specificity of memory B cells recalled by egg-grown influenza virus vaccines, we generated MAbs from sorted PBs 7 days following vaccination. The transient PB populations are highly specific to the components of the vaccine and are recalled from preexisting memory B cells (15–17). We focused our studies on MAbs generated from subjects following vaccination with the 2009 monovalent pandemic H1N1 inactivated influenza virus vaccine (MIV) and the 2010 trivalent inactivated influenza virus vaccine (TIV). From the vaccine-induced PBs, we found that 75% of MAbs bound HA (Fig. S1A and B in the supplemental material). Notably, 27 MAbs generated from multiple subjects receiving either of the egg-grown vaccines bound all influenza virus strains tested (Fig. 1A; Table S1). To confirm these MAbs were specific to influenza viruses and not an artifact of vaccine preparation in eggs, we tested MAb binding to A/California/7/2009 H1N1 virus grown in eggs or in mammalian Madin-Darby canine kidney (MCDK) cells. Strikingly, these MAbs only bound to A/California/7/2009 grown in eggs, and not to virus propagated in mammalian cells (Fig. 1B and C), indicating these broadly reactive MAbs were binding to an egg-related antigen. Moreover, these broadly reactive MAbs bound to allantoic fluid from both uninfected eggs and A/California/7/2009 H1N1-infected eggs (Fig. 1D and E), indicating these MAbs were specific to an egg-associated antigen.

10.1128/mBio.00838-21.1 FIG S1 MAb binding to HA and egg-derived antigen. (A) Proportion of total MAbs binding to HA after vaccination with an egg-grown vaccine. Number in the center indicates number of MAbs tested. (B) Proportions of HA-binding MAbs per subject. Data are mean ± SD. (C) Proportion of subjects with detectable egg MAbs. Number in the center indicates number of subjects. (D and E) Serum was isolated from subjects with or without isolated egg-binding MAbs before and 14 to 21 days after vaccination. (D) Fold changes (represented as a percentage) in serum IgG binding to rHA from A/California/7/2009 H1N1. (E) Serum IgG binding to A/California/7/2009 rHA before and after vaccination. Lines connect responses from individual subjects. (F) Fold changes in serum HAI titers against A/California/7/2009 after vaccination. (D and F) Lines are the medians. Data were analyzed using a two-tailed Mann-Whitney test. (E) Data were analyzed using a two-tailed Wilcoxon matched-pairs signed-rank test. Download FIG S1, DOCX file, 0.2 MB. Copyright © 2021 Guthmiller et al. 2021 Guthmiller et al. https://creativecommons.org/licenses/by/4.0/ This content is distributed under the terms of the Creative Commons Attribution 4.0 International license.

10.1128/mBio.00838-21.3 TABLE S1 Study cohort demographics. Download Table S1, DOCX file, 0.01 MB. Copyright © 2021 Guthmiller et al. 2021 Guthmiller et al. https://creativecommons.org/licenses/by/4.0/ This content is distributed under the terms of the Creative Commons Attribution 4.0 International license.

Fifty percent of all subjects analyzed generated egg-specific-antibody responses (Fig. S1C), with 1 of the 5 subjects receiving the MIV and 6 of the 9 subjects receiving the TIV generating an egg-specific-antibody response (Table S1). Within the subjects that mounted an antibody response against this egg-derived antigen, 31% of MAbs generated specifically bound the egg-derived antigen (Fig. 1F). The range of antibodies per subject ranged from 8% to 58% of the vaccine-induced PBs that were isolated (Fig. 1G). Additionally, we found that subjects that had egg-reactive MAbs had a larger fold increase in serum IgG responses against uninfected allantoic fluid than subjects that did not have detectable MAbs against the egg-associated antigen (Fig. 1H). Despite this, subjects that mounted an antibody response against the egg-associated antigen had fold increases in serum IgG titers against A/California/7/2009 recombinant HA and hemagglutination inhibition (HAI) titers against A/California/7/2009 H1N1 that were similar to those in subjects that did not generate a PB response against the egg antigen (Fig. S1D to F). Together, these data indicate that some subjects generate an antibody response against an egg-derived antigen following influenza virus vaccination.

### Viruses grown in allantoic fluid, but not other parts of the egg, possess the egg antigen.

**FIG 2:**
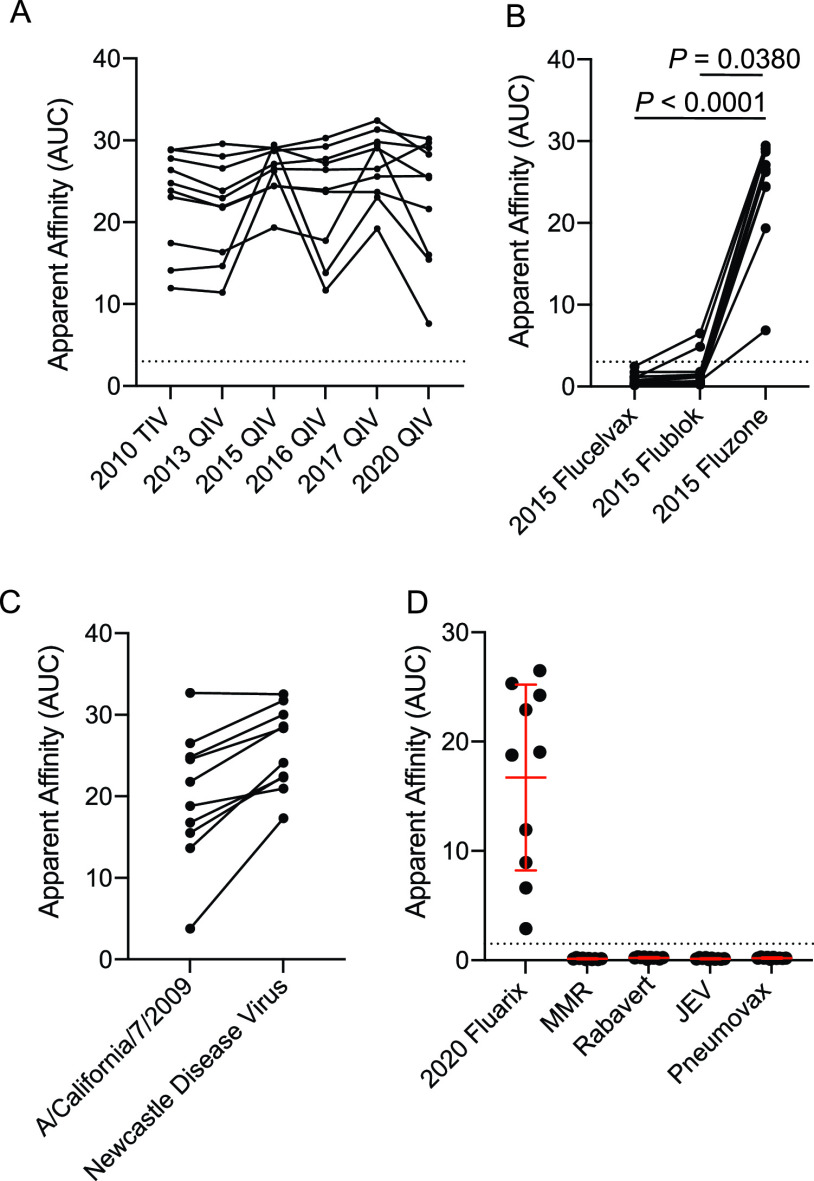
Binding is specific to viruses grown in eggs, and not chicken cells. (A) Apparent affinities (AUC) of egg-specific-MAb binding to influenza virus vaccines from multiple years. Each line connects the values for the same MAb binding different vaccines (*n* = 10 MAbs). (B) Apparent affinities of MAb binding to the mammalian cell-grown vaccine (Flucelvax), insect cell-grown vaccine (Flublok), and egg-grown vaccine (Fluzone). Each line connects the values for the same MAb binding different vaccines (*n* = 11 MAbs). (C) Egg-specific-MAb binding to Newcastle disease virus grown in eggs and egg-grown A/California/7/2009 H1N1 virus (*n* = 10). Each line connects the same MAb. (D) Egg-specific-MAb (*n* = 10) binding to the 2020/2021 Fluarix vaccine (egg-grown), measles/mumps/rubella vaccine (MMR; chicken cell line grown), RabAvert (rabies vaccine; chicken cell grown), Japanese encephalitis virus vaccine (JEV) (Ixiaro; Vero cell grown), and Pneumovax 23 vaccine (polysaccharides from bacteria). (D) Data are mean values ± SD. (B) Data were analyzed using a nonparametric Friedman test. (C) Data were analyzed using a two-tailed Wilcoxon matched-pairs signed-rank test.

Starting in 2018, the United States Centers for Disease Control and Prevention began recommending that people with egg allergies could receive egg-grown influenza virus vaccines, suggesting the major egg allergens were removed from the vaccine (https://www.cdc.gov/flu/prevent/egg-allergies.htm). Although subjects within our cohorts had not experienced an allergic response to influenza virus vaccination or reported a history of egg allergies, we next tested whether the identified egg-specific MAbs could bind to more recent inactivated influenza virus vaccines grown in eggs that lack the egg allergens. Notably, the egg-specific MAbs could bind old TIVs and quadrivalent inactivated influenza virus vaccines (QIVs) to a similar degree as they did recent egg-grown QIVs, including the 2020 Fluarix QIV (Fig. 2A), indicating that the egg-specific antigen identified still persists in recent egg-grown vaccines. We did observe some variability in MAb binding across different vaccine formulations, which may have been a result of vaccines being acquired from different manufacturers and having different viral formulations (Table S3). Additionally, some QIVs are also made from viruses grown in mammalian MDCK cells (Flucelvax) and recombinant HA generated in insect cells (Flublok). The egg-binding MAbs specifically bound to QIV viruses grown in eggs, but not viruses grown in MDCK or recombinant HA grown in insect cells (Fig. 2B). We also confirmed that these egg-binding MAbs could bind other viruses grown in the allantoic fluid of eggs, as these MAbs bound as strongly to Newcastle disease virus (NDV) grown in eggs as they did to A/California/7/2009 H1N1 grown in eggs (Fig. 2C). To understand how ubiquitous the egg antigen was across chicken cell- and egg-grown vaccines, we next tested for MAb binding to other vaccines grown in chicken cells. Notably, the mumps and measles viruses of the measles/mumps/rubella vaccine (MMR) are both grown in a chicken embryo cell line and the rabies virus in RabAvert is grown in primary chicken fibroblasts. As a control, we also tested the egg-binding MAbs for binding to non-egg- or chicken-grown vaccines, including the Japanese encephalitis virus vaccine (Ixiaro) grown in Vero cells and the Pneumovax 23 vaccine that contains purified capsular polysaccharides from 23 distinct Streptococcus pneumoniae serotypes. The egg-binding MAbs only bound to vaccines grown in eggs, and not to vaccines grown in a chicken embryo cell line (MMR), primary chicken fibroblasts (RabAvert), or vaccines not produced in eggs (Ixiaro and Pneumovax 23) (Fig. 2D). Together, these data reveal that the egg-associated antigen is only present in viruses grown in allantoic membrane, and not in those grown in cells isolated from chicken embryos or chicken fibroblasts.

10.1128/mBio.00838-21.5 TABLE S3 Vaccines used in study to test egg-MAb binding potential. Download Table S3, DOCX file, 0.01 MB. Copyright © 2021 Guthmiller et al. 2021 Guthmiller et al. https://creativecommons.org/licenses/by/4.0/ This content is distributed under the terms of the Creative Commons Attribution 4.0 International license.

### Antiegg antibodies bind a sulfated LacNAc.

**FIG 3:**
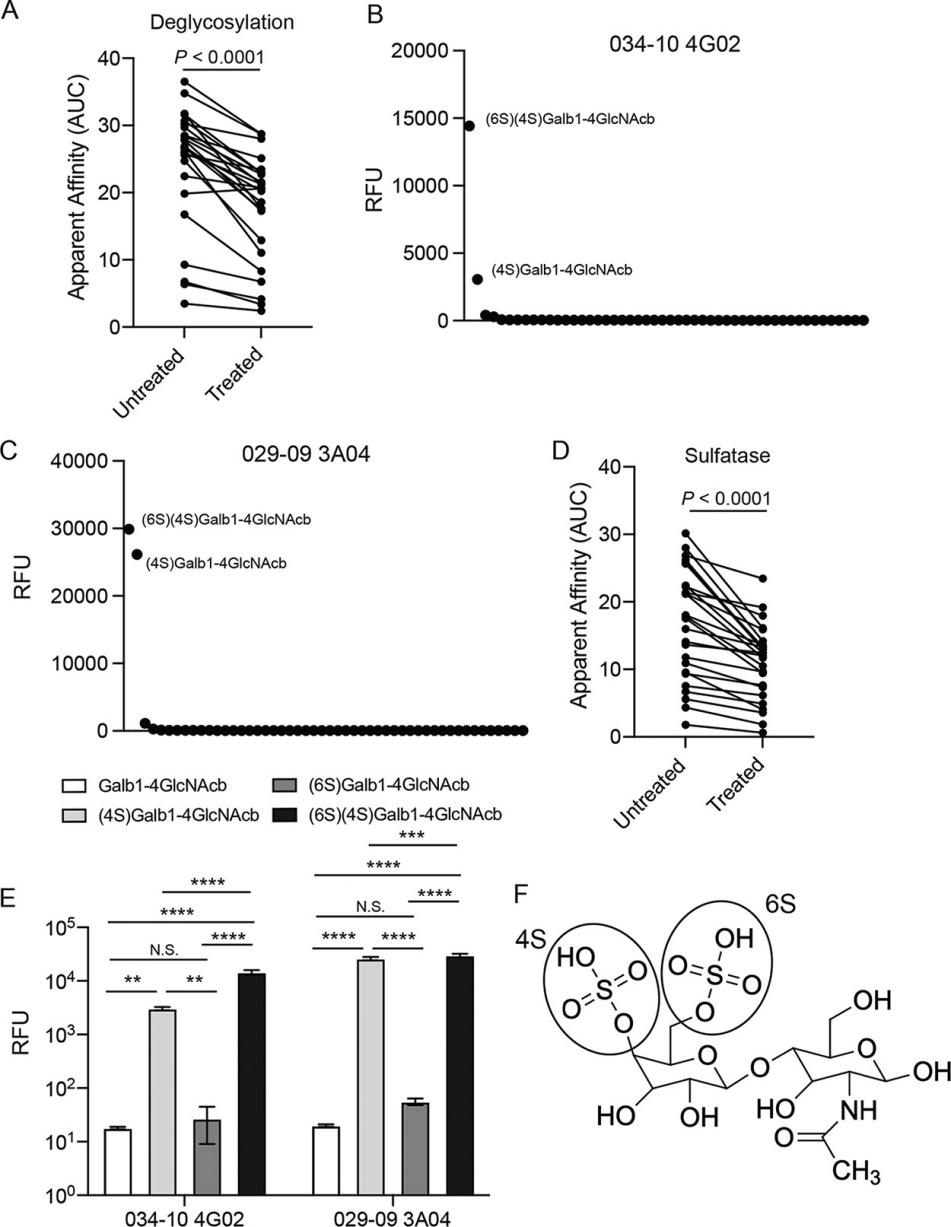
Egg-specific MAbs are binding to a sulfated LacNAc. (A) Egg-specific-MAb (*n* = 24) binding to deglycosylated or untreated 2020 Fluarix QIV. (B and C) MAbs 034-10 4G02 (B) and 029-09 3A04 (C) were tested for binding to glycans on a microarray. Data represent the top 50 glycan hits. (D) Egg-specific-MAb (*n* = 26) binding to sulfatase-treated or untreated A/Hong Kong/485197/2014 H3N2. (E) MAb 034-10 4G02 and 029-09 3A04 binding to nonsulfated and sulfated LacNAc glycans in glycan microarray. (F) Structure of (6*S*)(4*S*)Galβ1-4GlcNacβ (LacNAc). (B, C, and E) Data are averaged relative fluorescence unit (RFU) values from 4 replicates. Data are mean values ± SD. (A and D) Each line connects values for the same MAb. Data were analyzed using a two-tailed Wilcoxon matched-pairs signed-rank test. (E) Data were analyzed using an ordinary two-way analysis of variance (ANOVA).

Egg allergies are typically caused by antibody responses against ovalbumin and ovomucoid (18), which are present in both the egg white and allantoic fluid (19, 20). However, the egg-specific MAbs did not bind to ovalbumin or ovomucoid purified from egg whites (data not shown), further indicating that these MAbs were likely not specific to known egg allergens. As the antigen did not seem to be protein in nature, we next tested whether the egg-binding MAbs were binding an egg-associated glycan. To test this, we deglycosylated the 2020 Fluarix QIV with a deglycosylating enzyme that removes N-linked glycans. The MAbs had reduced binding to deglycosylated vaccine relative to their binding to untreated vaccine (Fig. 3A). To investigate the particular glycan these MAbs were binding, we tested two MAbs (029-09 3A04 and 034-10 4G02) for binding to a glycan microarray that included 585 distinct glycans (Tables S4 and S5). Both MAbs specifically bound to two sulfated LacNAc antigens, (6*S*)(4*S*)Glaβ1-4GlcNAcβ and (4*S*)Glaβ1-4GlcNAcβ (Fig. 3B and C). Treatment of purified egg-grown A/Hong Kong/485197/2014 H3N2 with a sulfate ester sulfatase significantly reduced egg-specific-MAb binding (Fig. 3D). Moreover, both MAbs only bound to LacNAc with a sulfate group on the hydroxyl group of 4C′ of galactose, and not the hydroxyl group on 6C′ of the galactose of LacNAc only or an unsulfated LacNAc (Fig. 3E and F; Tables S4 and S5). Together, these data reveal that B cells induced by influenza viruses grown in eggs are binding a sulfated LacNAc.

10.1128/mBio.00838-21.6 TABLE S4 Glycans in microarray stratified by RFU for 034-10 4G02. In attached Excel sheet. Download Table S4, XLSX file, 0.04 MB. Copyright © 2021 Guthmiller et al. 2021 Guthmiller et al. https://creativecommons.org/licenses/by/4.0/ This content is distributed under the terms of the Creative Commons Attribution 4.0 International license.

10.1128/mBio.00838-21.7 TABLE S5 Glycans in microarray stratified by RFU for 029-09 3A04. In attached Excel sheet. Download Table S5, XLSX file, 0.04 MB. Copyright © 2021 Guthmiller et al. 2021 Guthmiller et al. https://creativecommons.org/licenses/by/4.0/ This content is distributed under the terms of the Creative Commons Attribution 4.0 International license.

### Egg-binding antibodies utilize a restricted repertoire and resemble natural antibodies.

**FIG 4:**
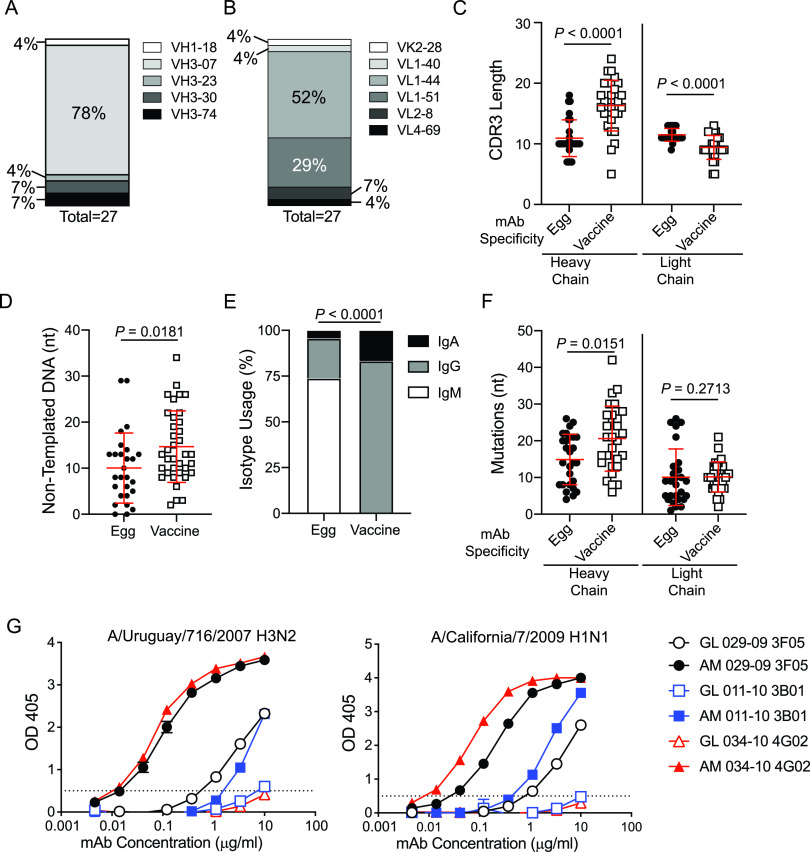
Egg-binding MAbs resemble natural antibodies. (A and B) V_H_ (A) and V_K_/V_L_ (B) gene usage of egg-binding MAbs. (C and D) Heavy-chain and light-chain CDR3 lengths (C) and nontemplated DNA, or N-nucleotides (D), of egg-binding- and vaccine-specific-MAb heavy chains. (E and F) Isotype usage (E) and somatic hypermutations (nucleotide mutations [nt]) (F) of egg-binding and vaccine-specific MAbs. (G) Affinity-matured (AM) 029-09 3F05, 011-10 3B01, and 034-10 4G02 were reverted back to germ line (GL) and were tested for binding to egg-grown A/Uruguay/716/2007 H3N2 and A/California/7/2009 H1N1 relative to their affinity-matured counterparts. (C, D, F, and G) Data are mean values ± SD. (C, D, and F) Data were analyzed using two-tailed Mann-Whitney tests. (E) Data were analyzed using chi-squared test.

Of the egg-binding MAbs, we identified strong repertoire biases on the usage of particular heavy- and light-chain variable genes, with the vast majority of MAbs using V_H_3-07 and V_L_1-44 (Fig. 4A and B). However, there was a lot of diversity in the H-CDR3 sequences, with no consensus on D_H_ gene usage (Fig. S2A). H-CDR3s and light-chain CDR3s (L-CDR3s) of egg-binding MAbs preferentially used J_H_4 and J_L_3, suggesting there was some selection for certain J genes (Fig. S2B and C). Moreover, H-CDR3s of egg-binding MAbs were significantly shorter than those of vaccine-specific MAbs, whereas the L-CDR3s were significantly longer than vaccine-specific MAbs (Fig. 4C). Concordantly, H-CDR3s of egg-binding MAbs had fewer nontemplated DNA insertions, or N-nucleotides, than did vaccine-specific MAbs (Fig. 4D; Fig. S2D).

10.1128/mBio.00838-21.2 FIG S2 Additional repertoire and clonal expansions of egg-binding MAbs. (A to C) D_H_ (A), J_H_ (B), and J_κ_/J_L_ (C) gene usage by egg-binding MAbs. (D) N1 (V-D) and N2 (D-J) nontemplated nucleotide insertions of heavy-chain CDR3s of egg- and vaccine-binding MAbs. (E) Clonal relatedness of heavy chains and light chains of egg-binding MAbs. (F) Polyreactivities of egg-binding MAbs relative to vaccine-specific MAbs. (G to I) Expansion of an egg-binding clone across multiple years of vaccination. (G) Alignment of heavy-chain and light-chain sequences. (H and I) Binding curves of MAbs binding to A/California/7/2009 (H) and apparent affinities (AUC) (I) of MAbs binding to egg-grown influenza viruses. (D and H) Data are mean values ± SD. (D) Data were analyzed using two-tailed Mann-Whitney tests. (F) Data were analyzed using chi-square test. (I) Data were analyzed using nonparametric paired Friedman tests. Download FIG S2, DOCX file, 0.4 MB. Copyright © 2021 Guthmiller et al. 2021 Guthmiller et al. https://creativecommons.org/licenses/by/4.0/ This content is distributed under the terms of the Creative Commons Attribution 4.0 International license.

Analysis of clonal expansions revealed that the light chains of egg-binding MAbs were highly clonal across individual MAbs, with one light-chain clone occupying over one-third of all egg-binding MAbs (Fig. S2E; Tables S2 and S6). Additionally, we identified four distinct clonal expansions, accounting for one-third of all antibodies identified (Fig. S2E). However, these paired heavy- and light-chain clones were private, with individual clones only identified in one subject each (Table S2). PBs induced by vaccination derive from memory B cells and, therefore, are usually class-switched to IgG and highly mutated (17). However, egg-binding MAbs were largely IgM and had fewer mutations in the heavy chain than did vaccine-specific MAbs (Fig. 4E and F). Notably, clonal expansions of egg-binding MAbs that utilized IgM and IgG were observed (Table S2), suggesting the same clonal expansion could class switch. In combination, egg-binding MAbs resemble natural antibodies produced by innate B cells, as they express simple and short H-CDR3s, do not commonly class switch, and have fewer mutations than vaccine-specific antibodies (21, 22). In addition, natural antibodies commonly target glycans (23) and are typically polyreactive (24, 25). However, egg-binding MAbs were not enriched for polyreactivity, as measured by binding to unrelated antigens (lipopolysaccharide [LPS], cardiolipin, insulin, double-stranded DNA [dsDNA], flagellin, and keyhole limpet hemocyanin [KLH]), relative to the polyreactivity of vaccine-induced antibodies (Fig. S2F). Despite having fewer mutations than vaccine-induced antibodies, germ line (GL)-reverted egg-binding MAbs had reduced binding affinity for influenza viruses grown in eggs relative to that of the affinity-matured (AM) MAbs generated from PBs (Fig. 4G). Furthermore, we identified a clonal expansion from one subject over two influenza virus vaccine seasons (2010 TIV and 2011 TIV), with the MAbs from 2011 having higher affinity for influenza virus strains than the MAbs from 2010 (Fig. S2G to I). With the highly restricted V_H_/V_L_ repertoire, short H-CDR3 sequences and reduced N-nucleotide additions, lack of class switching, and fewer mutations, MAbs targeting the egg antigen resemble natural antibodies produced by innate-like B cells (26).

10.1128/mBio.00838-21.4 TABLE S2 Egg-binding MAb information. Clonal number of 0 indicates a particular chain was nonclonal. Download Table S2, DOCX file, 0.02 MB. Copyright © 2021 Guthmiller et al. 2021 Guthmiller et al. https://creativecommons.org/licenses/by/4.0/ This content is distributed under the terms of the Creative Commons Attribution 4.0 International license.

10.1128/mBio.00838-21.8 TABLE S6 Heavy- and light-chain clonal information. Download Table S6, DOCX file, 0.01 MB. Copyright © 2021 Guthmiller et al. 2021 Guthmiller et al. https://creativecommons.org/licenses/by/4.0/ This content is distributed under the terms of the Creative Commons Attribution 4.0 International license.

### Influenza virus vaccines commonly induce PBs with repertoire features of egg-binding MAbs.

**FIG 5:**
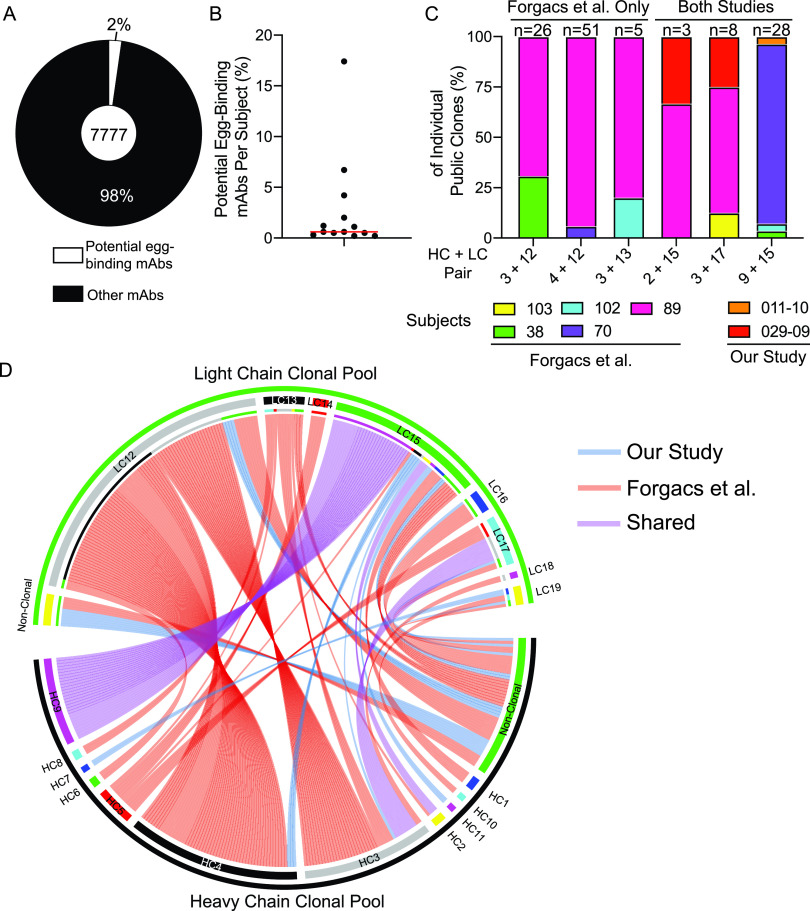
PBs with repertoire features of egg-binding antibodies are common after vaccination. B cell receptor sequences from 7,777 IgG^+^ PBs induced by influenza virus vaccination were analyzed for repertoire features of egg-binding MAbs (V_H_3-7 with H-CDR3 of ≤12 amino acids paired with V_L_1-44 or V_L_1-51). (A and B) Proportions of sequences with egg-binding MAb repertoire features out of all sequences (A) and by subject (B). (B) Red line represents the median. (C) Subject makeup (%) of public clones, including public clones specific to Forgacs et al. (27) and shared across studies. The number above each column represents the number of clonal members per clone. (D) Circos plot of heavy- and light-chain clones distinct to each study or shared across studies.

We next addressed whether antibodies with repertoire features of the egg-binding antibodies are commonly induced after influenza virus vaccination. To dissect this question, we utilized a data set of 7,777 B cell receptor sequences from influenza virus vaccine-induced PBs from subjects that received the egg-grown 2016–2017 Fluzone QIV (27). From this data set, we identified that 2% (175 total) of all IgG-expressing (IgG^+^) PBs expressed V_H_3-7 with an H-CDR3 length equal to or less than 12 amino acids that was paired with V_L_1-44 or V_L_1-51 (potential egg-binding MAbs) (Fig. 5A). Notably, 13 of 17 total subjects had PBs with these repertoire features, occupying 0.2 to 17.4% of the PB response per subject (Fig. 5B). Notably, this data set was specifically generated from IgG^+^ PBs. As we determined that most egg-binding MAbs were IgM (Fig. 4E), the true number of potential egg-specific PBs induced within these subjects may be substantially higher. Of the 175 heavy- and light-chain pairings identified, we discovered 6 public clonotypes (Fig. 5C; Table S6), which comprised 66.9% of the total response (117/175 paired sequences). Strikingly, 3 of the public clones were shared between our study and the data set (Fig. 5C; Table S6), suggesting that the PBs induced in subjects in the Forgacs et al. study (27) are specific to the egg glycan. Moreover, we identified that egg-binding MAbs from our study shared at least a heavy-chain or light-chain clone with the potential egg-binding PBs from the IgG^+^ PB data set (Fig. 5D; Table S6). Together, these data suggest that PBs targeting an egg-associated glycan are commonly induced by influenza virus vaccines grown in eggs.

## DISCUSSION

In this report, we identified that egg-grown viruses possess an egg-associated glycan that is immunogenic in humans. Egg-specific MAbs from PBs had evidence of prior affinity maturation and likely derived from memory B cells that were primed by earlier vaccination with egg-grown vaccines. Moreover, most subjects within the 2010 TIV cohort had previously been vaccinated with the 2009 MIV, suggesting that prior exposure to the egg antigen from the 2009 MIV generated memory B cells against this antigen. Despite some subjects mounting a response against the egg antigen, the same subjects seroconverted against the H1N1 component of the vaccine to similar levels as subjects that did not mount a response against the egg-associated antigen. Moreover, 13 of 17 subjects from Forgacs et al. had detectable PBs with repertoire features of egg-binding MAbs. Notably, this study specifically recruited subjects that had not been vaccinated in the prior 3 seasons (27). Prior research has indicated that preexisting serum antibodies against the strains included in the vaccine inversely correlate with the induction of PBs by vaccination (28). Therefore, subjects that mounted an antiegg response perhaps had more B cell activation than subjects that did not and as a result had similar anti-HA antibody responses.

Despite no differences in serum antibodies against the H1N1 component of the vaccine, B cells mounted against the egg antigen may compete within germinal centers, with B cells targeting protective epitopes of HA and NA, which could perturb the generation of plasma cells and memory B cells against protective epitopes. In accordance, we identified that the same egg-specific clone was recalled over multiple vaccine years, suggesting that subjects can repeatedly recall memory B cells against the egg-derived antigen upon repeated vaccination and further indicating that egg-specific antibodies are fixed in the memory B cell repertoire. Therefore, our study suggests that repeatedly vaccinated subjects can preferentially recall memory B cells targeting irrelevant antigens associated with egg-based vaccine production, which could come at the cost of affinity maturation and generation of plasma cells and memory B cells specific for protective epitopes that provide long-lived protection against influenza viruses. However, the precise role of egg-specific B cells competing with virus-specific B cells remains to be determined. Our study is too underpowered to make any conclusions on whether the antiegg response in some subjects affects the protective antibody response against influenza viruses. Importantly, future studies focused on understanding the impact of antiegg B cells on the generation of protective antibody responses against influenza viruses are critically important, as this question informs vaccine preparation and manufacturing practices.

The egg-binding MAbs demonstrated features of natural antibodies produced by innate-like B cells, including their glycan specificity and repertoire features. Natural antibodies are typically raised against self-antigens and evolutionarily conserved antigens like lipids and glycans (29). In humans, natural antibodies are largely elicited against glycans, including the blood group A and B antigens and xenoglycans from other mammals, such as the “α-gal” epitope of galactose-α-1,3-galactose expressed by most nonprimate mammals (30, 31). Our studies reveal that the egg-binding antibodies were specifically targeting a secondary sulfate structure of LacNAc, a common glycan found across all life forms. Although the egg-binding MAbs identified in this study share key characteristics of natural antibodies, the precise cellular origins of these antibodies require further analysis. Moreover, whether all antibodies identified in our study bind this sulfated LacNAc is yet to be determined. As the anti-sulfated-LacNAc MAbs were confirmed from two subjects, possess similar repertoire features, and have reduced binding to deglycosylated and sulfatase-treated virus, the antiegg antibodies likely bind the same or a similar glycan found in eggs.

LacNAcs are a critical component of glycosaminoglycans (GAGs), including keratan sulfate and the Lewis blood group determinants (32, 33). LacNAcs are also the main ligand for galectins and mediate a variety of cellular functions, including cell adhesion, migration, proliferation, and apoptosis (34–36). However, most mammals do not commonly sulfonate the galactose 4C′ of LacNAc, but instead commonly sulfate the 6C′ of galactose of LacNAc and 6C′ of *N*-acetylglucosamine of LacNAC (37). Therefore, humans may mount a response specifically against the sulfated 4C′ of LacNAc, as it is not normally sulfated in humans. However, inflammation is associated with aberrant glycosylation patterns, including during cancer and autoimmunity (38). Moreover, antibodies against a sulfated 4C′ LacNAc, the same antigen identified in this study, were elevated in subjects with systemic sclerosis and were associated with a higher prevalence of pulmonary hypertension (39). Despite these findings, the precise role and function of anti-sulfated-LacNAc antibodies in the development and severity of systemic sclerosis remain unknown. Furthermore, it is unknown if the antibodies induced by egg-grown influenza virus vaccines and those observed during systemic sclerosis share similar repertoire features and could be derived from the same B cell precursors. Lastly, it remains unknown how the 4C′ sulfate LacNAc is conjugated to influenza viruses grown in eggs, as a study of the glycosylation patterns of HA and NA isolated from egg-grown viruses did not have this glycan (40). Moreover, the precise antigen in eggs remains unknown. In summary, this study identifies that antibodies with features of natural antibodies can be induced against a sulfated LacNAc glycan present in egg-grown vaccines.

## MATERIALS AND METHODS

### Monoclonal antibody production and sequence analysis.

Monoclonal antibodies were generated as previously described (15, 41, 42). Peripheral blood was obtained from each subject approximately 7 days after vaccination or infection. Lymphocytes were isolated and enriched for B cells using RosetteSep. PBs (CD3^−^ CD19^+^ CD27^hi^ CD38^hi^) were single cell sorted into 96-well plates. Immunoglobulin heavy- and light-chain genes were amplified by reverse transcriptase PCR (RT-PCR), sequenced, cloned into human IgG1, human kappa chain, or human lambda expression vectors, and cotransfected into HEK293T cells. Secreted MAbs were purified from the supernatant using protein A-agarose beads. B cell clones were determined by aligning all the V(D)J sequences sharing identical progenitor sequences, as predicted by IgBLAST using our in-house software, Vgenes. For germ line MAbs, germ line sequences were synthesized (IdT) and cloned into antibody expression vectors as described above.

### Antibody sequences and clonal analyses.

Previously published IgG^+^ PB sequences (27) were downloaded from NCBI GenBank (KEOV00000000 and KEOU00000000). V(D)J gene usage from our study and Forgacs et al. (27) were analyzed using IgBlast, and clones were determined using our in-house software, Vgenes, based on germ line sequences. For identification of egg-like MAbs from Forgacs et al., we selected B cell clones that specifically used V_H_3-7 with an H-CDR3 of 12 or fewer amino acids that was paired with V_L_1-44 or V_L_1-51. MAb sequence alignments were made using ClustalOmega (EMBL-EBI). Nontemplated-nucleotide insertions at the V-D and D-J junctions of the heavy-chain gene were identified using partis version 0.15.0, a hidden Markov model-based tool for annotating B cell receptor sequences (43). Custom code was used for processing the output (available at https://github.com/cobeylab/egg_antibodies). The visualization of clones presented in Fig. 5D was performed in R using circlize version 0.4.12 (44).

### Viruses, proteins, and vaccines.

Influenza viruses used in all assays were grown in-house in specific-pathogen-free (SPF) eggs, harvested and purified, and the titers determined. Allantoic fluid was harvested from both infected and uninfected eggs. For MDCK cell-grown virus, A/California/7/2009 H1N1 was grown in MDCK-SIAT1 cells, concentrated, and chemically inactivated with beta-propiolactone. Newcastle disease virus was grown in eggs, and allantoic fluid was harvested and subsequently inactivated with beta-propiolactone, purified, and quantified. Vaccines used for MAb binding assays are outlined in Table S3. Recombinant HA (rHA) from A/California/7/2009 was expressed in HEK293T cells.

### Antigen-specific ELISA.

High-protein-binding microtiter plates (Costar) were coated with 8 hemagglutination units (HAU) of virus or allantoic fluid diluted 1:500 in carbonate buffer overnight at 4°C. The plates were coated with recombinant HA from A/California/7/2009 at 1 μg/ml in phosphate-buffered saline (PBS) overnight at 4°C. For testing egg-binding-MAb binding to various vaccines, influenza virus vaccines were diluted to 5 μg/ml, RabAvert was diluted to 0.05 IU/ml, MMR was diluted 1:100, Ixiaro (JEV) was diluted to 0.05 antigen units per ml, and Pneumovax 23 was diluted to 5 μg/ml. All vaccines tested were diluted in PBS and coated overnight at 4°C. NDV was diluted to 5 μg/ml in carbonate buffer, and plates were coated overnight at 4°C.

Plates were washed the next morning with PBS/0.05% Tween and blocked with PBS containing 20% fetal bovine serum (FBS) for 1 h at 37°C. MAbs were then serially diluted 3-fold starting at 10 μg/ml and incubated for 1.5 h at 37°C. For serum enzyme-linked immunosorbent assays (ELISAs), serum was diluted 1:50 and further diluted 2-fold. Horseradish peroxidase (HRP)-conjugated goat anti-human IgG antibody diluted 1:1,000 (Jackson ImmunoResearch) was used to detect binding of MAbs and serum antibodies, and plates were subsequently developed with super aquablue ELISA substrate (eBiosciences). Absorbance was measured at 405 nm on a microplate spectrophotometer (Bio-Rad). To standardize the assays, control antibodies with known binding characteristics were included on each plate, and the plates were developed when the absorbance of the control reached 3.0 optical density (OD) units. For the other vaccines used in the experiment whose results are shown in Fig. 2D, antisera against the various vaccines were used to confirm the antigenicity of the vaccines. To determine MAb affinity, a nonlinear regression was performed on background-subtracted ODs, and area under the curve (AUC) values were reported. Serum samples used in the experiments whose results are shown in Fig. 1H and Fig. S1D to F are listed in Table S7. All MAbs or serum samples were tested in duplicate, and all assays were performed 2 or 3 times.

10.1128/mBio.00838-21.9 TABLE S7 Serum donors for samples analyzed in Fig. 1H and Fig. S1D to F. Download Table S7, DOCX file, 0.01 MB. Copyright © 2021 Guthmiller et al. 2021 Guthmiller et al. https://creativecommons.org/licenses/by/4.0/ This content is distributed under the terms of the Creative Commons Attribution 4.0 International license.

### Polyreactivity ELISAs.

Polyreactivity was determined using a polyreactive ELISA protocol, as previously described (13). Briefly, MAbs were tested for binding to 6 antigens (cardiolipin, dsDNA, flagellin, insulin, KLH, and LPS) starting at 1 μg/ml for 1.5 h at 37°C. HRP-conjugated goat anti-human IgG antibody (Jackson ImmunoResearch) diluted 1:2,000 in PBS/0.05% Tween/0.1 mM EDTA was used to detect binding of MAbs, and plates were subsequently developed with super aquablue ELISA substrate (eBiosciences). MAbs were considered polyreactive if they bound 4 or more antigens at an OD of 450 nm (OD_450_) of ≥0.5. All MAbs were tested in duplicate, and all assays were performed 2 or 3 times.

### HAI assays.

For serum HAI assays, 1 part serum was treated with 3 parts receptor-destroying enzyme II (Seiken, Hardy Diagnostics) for 18 h at 37°C, followed by 30 min at 56°C. Serum was further diluted to 1:10 with PBS and serially diluted 2-fold in PBS in duplicate in a 96-well round-bottom plate. Serially diluted serum was mixed with an equal volume of A/California/7/2009 virus (4 HAU/25 μl) and subsequently incubated at room temperature for 1 h. Fifty microliters of 0.5% turkey red blood cells (Lampire Biological) was added to each well and incubated for 45 min at room temperature. HAI titers were determined based on the final dilution of serum for which hemagglutination inhibition was observed. All experiments were performed in duplicate twice. The fold changes in HAI serum titers of postvaccination samples relative to those of prevaccination samples are shown in Fig. S1F.

### Virus deglycosylation and sulfatase treatment.

To deglycosylate the vaccine, 25 μg of the 2020 QIV was denatured for 10 min at 75°C and treated with protein deglycosylation mix II (New England Biolabs) for 30 min at 25°C and 1 h at 37°C. For sulfatase treatment, A/Hong Kong/485197/2014 H3N2 virus was diluted to 160 HAU in sodium acetate (pH 5.0) and treated with 20 units/ml of sulfatase from abalone entrails (Sigma-Aldrich) for 1 h at 37°C. As a control, equal quantities were diluted and incubated but did not receive the deglycosylation mix II or sulfatase enzymes and are referred to as untreated. After treatment, preparations underwent buffer exchange with PBS to remove freed glycans and sulfate groups. ELISA plates were coated at 1 μg/ml for the 2020 QIV or 8 HAU for the virus.

### Glycan microarray.

MAbs 029-09 3A04 and 034-10 4G02 were sent to the Protein-Glycan Interaction Resource of the Center for Functional Glycomics at the Beth Israel Deaconess Medical Center, Harvard Medical School. Printed arrays consist of 585 glycans in replicates of 6. All glycans used in the microarray are listed in Tables S4 and S5. MAbs were diluted to 50 μg/ml and run on the array. The replicates with the highest and lowest values from each set of 6 replicates were removed, and the average value ± standard deviation (SD) was calculated from the middle 4 replicates. The structure of 6*S*,4*S*-LacNAc was made using ChemDraw JS (PerkinElmer).

### Statistics.

All statistical analysis was performed using Prism software (GraphPad version 9.0). *P* values less than or equal to 0.05 were considered significant. *P* values are indicated in figures as follows: *, *P* ≤ 0.05; **, *P* ≤ 0.01; ***, *P* ≤ 0.001; ****, *P* < 0.0001; N.S., nonsignificant. The specific numbers of MAbs shown in each figure are listed in the corresponding figure legends.

### Subjects, cohorts, and study approval.

Written informed consent was received from participants prior to inclusion in the study. All studies were performed with the approval of the University of Chicago and Emory University institutional review boards. Subjects were recruited to receive the Sanofi Pasteur 2009 pandemic H1N1 MIV or the 2010 Novartis Fluvirin TIV. Subjects labeled with SFV were recruited and vaccinated at Emory University, as previously described (45). All other subjects were recruited and vaccinated at the University of Chicago. Subject demographics are detailed in Table S1.

### Data availability.

Heavy and light chain sequences for the egg-binding MAbs used in this study were deposited in GenBank (MZ286600–MZ286626 and MZ297249–MZ297275).
