# Supplementary material for: An Egg-Derived Sulfated N-Acetyllactosamine Glycan Is an Antigenic Decoy of Influenza Virus Vaccines
Source: mBio. 2021 Jun 15;12(3):e00838-21. doi: 10.1128/mBio.00838-21 (PMC8263001; doi:10.1128/mBio.00838-21)
Supplement: FIG S1 [file mbio.00838-21-sf001.docx]

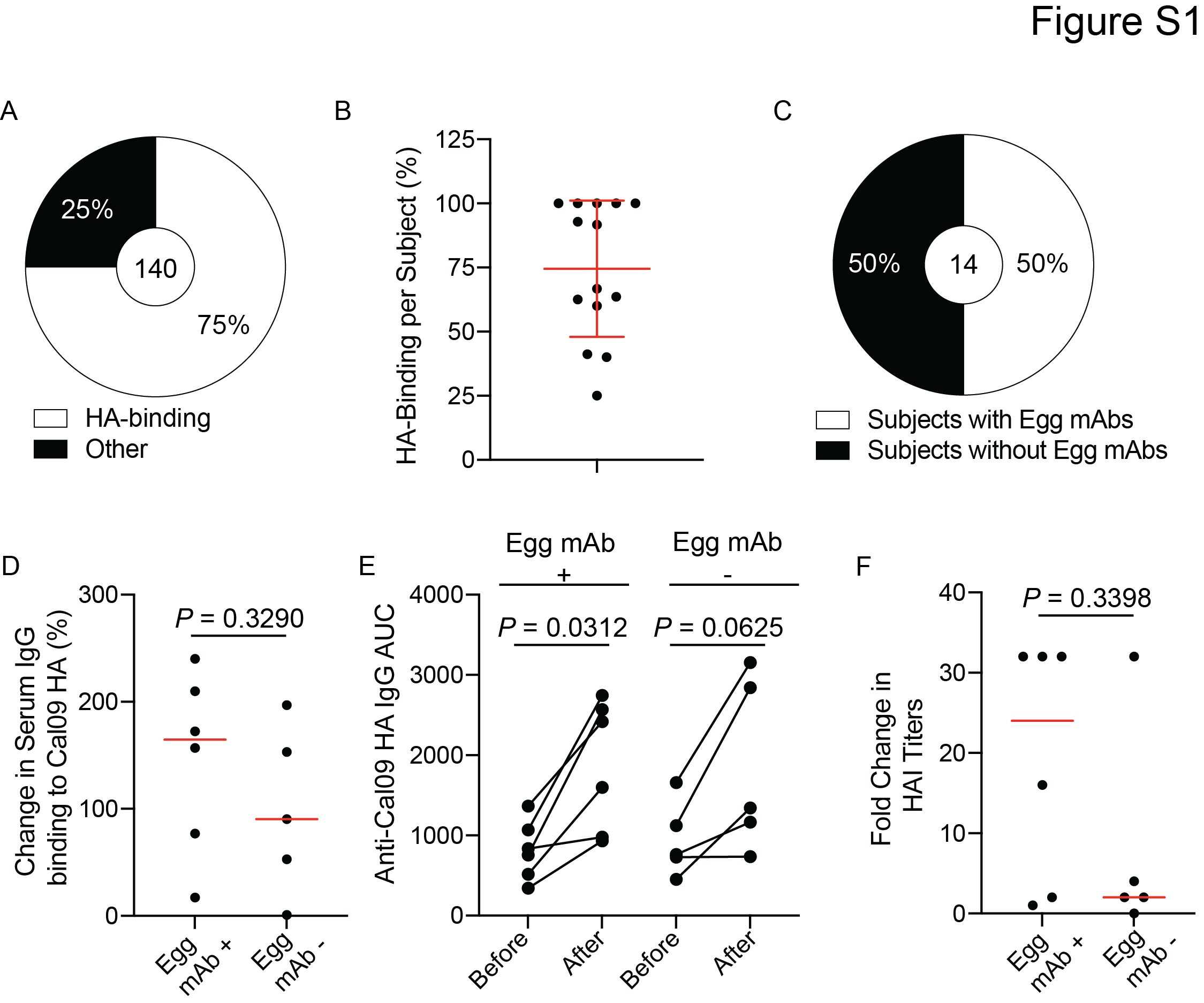


**Figure S1: MAb binding to HA and egg-derived antigen. A**, Proportion of total mAbs binding to HA after vaccination with an egg-grown vaccine. Number in the center indicates number of mAbs tested. **B**, Proportion of HA-binding mAbs per subjects. Data are mean ± S.D. **C**, Proportion of subjects with detectable egg mAbs. Number in the center indicates number of subjects. **D** and **E**, Serum was isolated from subjects with or without isolated egg-binding mAbs before and 14-21 days after vaccination. **D**, Fold-change (represented as a percentage) in serum IgG binding to rHA from A/California/7/2009 H1N1. **E**, Serum IgG binding to A/California/7/2009 rHA before and after vaccination. Lines connect responses from individual subjects. **F**, Fold change in serum HAI titers against A/California/7/2009 after vaccination. Lines in **D** and **F** are the median. Data in **D** and **F** were analyzed using a two-tailed Mann-Whitney test.
