# Supplementary material for: An Egg-Derived Sulfated N-Acetyllactosamine Glycan Is an Antigenic Decoy of Influenza Virus Vaccines
Source: mBio. 2021 Jun 15;12(3):e00838-21. doi: 10.1128/mBio.00838-21 (PMC8263001; doi:10.1128/mBio.00838-21)
Supplement: TABLE S1 [file mbio.00838-21-st001.docx]

| **Egg Reactivity** | **Subject** | **Vaccine Year/**  **Formulation** | **Vaccine/**  **Manufacturer** | **Known Prior**  **Vaccination(s)** | **Age** | **Sex** |
| --- | --- | --- | --- | --- | --- | --- |
| Egg mAb  Positive | 029-09 | 2009 MIV | Sanofi Pasteur | Unknown | 24 | M |
|  | 008-10 | 2010 TIV | Novartis Fluvirin | 2009 TIV,  2009 MIV | 26 | F |
|  | 011-10 | 2010 TIV | Novartis Fluvirin | Unknown | 30 | M |
|  | 017-10 | 2010 TIV | Novartis Fluvirin | 2009 MIV | 24 | M |
|  | 019-10 | 2010 TIV | Novartis Fluvirin | 2009 MIV | 23 | F |
|  | 034-10 | 2010 TIV | Novartis Fluvirin | Unknown | 40 | F |
|  | 051-10 | 2010 TIV | Novartis Fluvirin | 2009 MIV | 43 | M |
| Egg mAb  Negative | 030-09 | 2009 MIV | Sanofi Pasteur | Unknown | 31 | F |
|  | SFV018 | 2009 MIV | Sanofi Pasteur | 2009 TIV | 58 | F |
|  | SFV019 | 2009 MIV | Sanofi Pasteur | 2009 TIV | 48 | F |
|  | SFV020 | 2009 MIV | Sanofi Pasteur | 2009 TIV | 64 | F |
|  | 014-10 | 2010 TIV | Novartis Fluvirin | 2009 TIV,  2009 MIV | 27 | M |
|  | 028-10 | 2010 TIV | Novartis Fluvirin | Unknown | 32 | M |
|  | 039-10 | 2010 TIV | Novartis Fluvirin | 2009 TIV,  2009 MIV | 25 | M |

**Table S1: Study cohort demographics.**
