# Supplementary material for: An Egg-Derived Sulfated N-Acetyllactosamine Glycan Is an Antigenic Decoy of Influenza Virus Vaccines
Source: mBio. 2021 Jun 15;12(3):e00838-21. doi: 10.1128/mBio.00838-21 (PMC8263001; doi:10.1128/mBio.00838-21)
Supplement: TABLE S3 [file mbio.00838-21-st003.docx]

| **Vaccine** | **Manufacturer/Name** | **Propagation Cell Type/Host** | **Target** |
| --- | --- | --- | --- |
| 2010 TIV | Novartis – Fluvirin | Egg | Influenza Viruses |
| 2013 QIV | GSK – Fluarix | Egg | Influenza Viruses |
| 2015 QIV | Sanofi – Fluzone | Egg | Influenza Viruses |
| 2015 QIV | Seqirus – Flucelvax | MDCK Cells - Mammalian | Influenza Viruses |
| 2015 QIV | Sanofi – Flublok | Insect cells | Influenza Viruses |
| 2016 QIV | GSK – Fluarix | Egg | Influenza Viruses |
| 2017 QIV | GSK – Fluarix | Egg | Influenza Viruses |
| 2020 QIV | GSK – Fluarix | Egg | Influenza Viruses |
| MMR | Merck – MMR-II | Chicken-derived cell line | Measles, Mumps, Rubella Viruses |
| Rabavert | GSK | Primary chicken fibroblasts | Rabies Virus |
| Ixiaro | Valneva | Vero cell line - Mammalian | Japanese Encephalitis Virus |
| Pneumovax-23 | Merck | *Streptococcus pneumoniae* | *Streptococcus pneumoniae* polysaccharides from 23 serotypes |

**Table S3: Vaccines used in study to test egg-mAb binding potential.**
