# Supplementary material for: An Egg-Derived Sulfated N-Acetyllactosamine Glycan Is an Antigenic Decoy of Influenza Virus Vaccines
Source: mBio. 2021 Jun 15;12(3):e00838-21. doi: 10.1128/mBio.00838-21 (PMC8263001; doi:10.1128/mBio.00838-21)
Supplement: FIG S2 [file mbio.00838-21-sf002.docx]

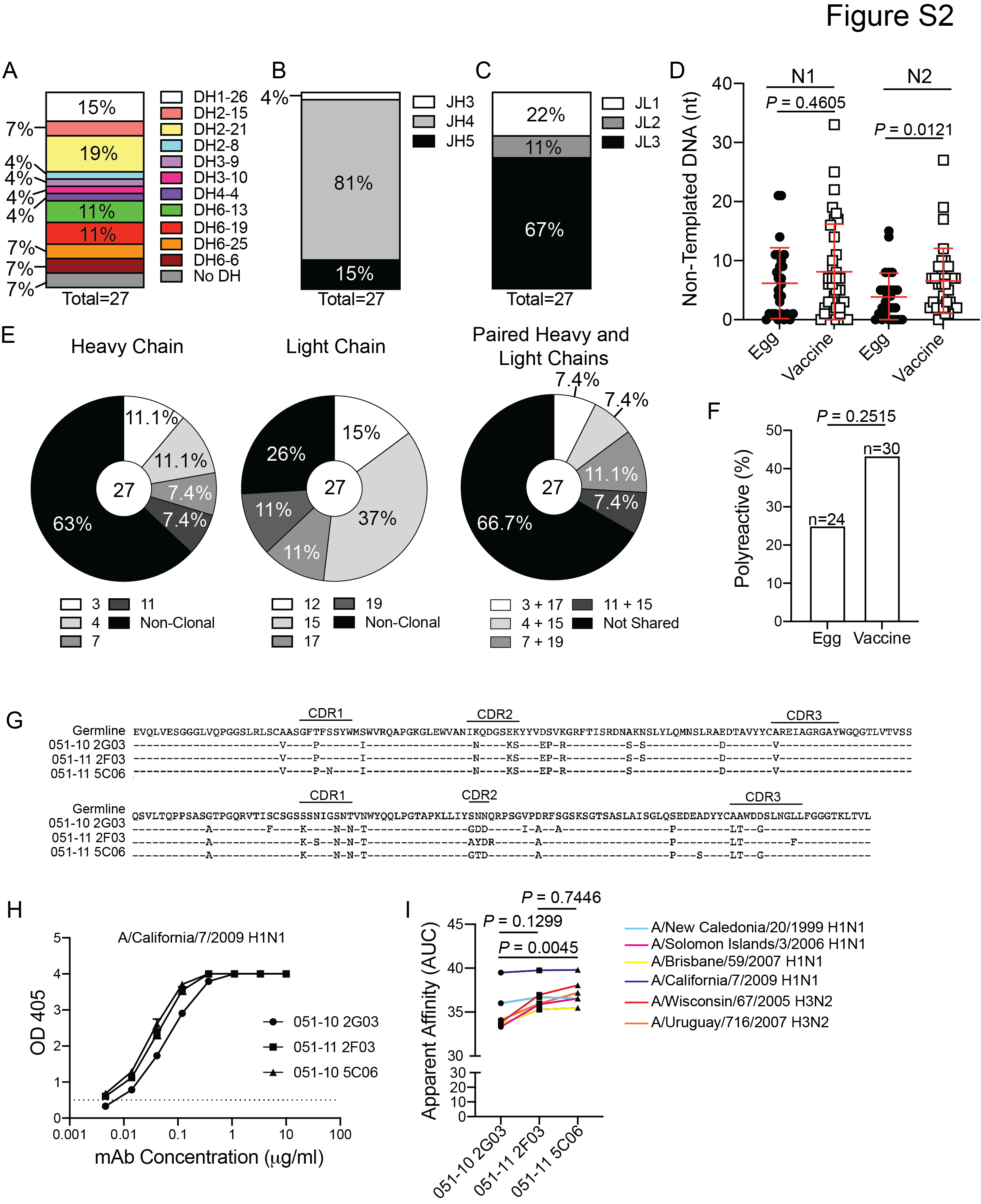


**Figure S2: Additional repertoire and clonal expansions of egg-binding mAbs. A**-**C**, DH (**A**), JH (**B**), and JK/JL (**C**) gene usage by egg-binding mAbs. **D**, N1 (V-D) and N2 (D-J) non-templated nucleotide insertions of heavy chain CDR3s of egg- and vaccine-binding mAbs. **E**, clonal relatedness of heavy chain and light chains of egg-binding mAbs. **F**, polyreacitivity of egg-binding mAbs relative to vaccine-specific mAbs. **G**-**I**, Expansion of an egg-binding clone across multiple years of vaccination. **G**, Alignment of heavy chain and light chain sequences. **H** and **I**, Binding curves of mAbs binding to A/California/7/2009 (**H**) and apparent affinity (AUC; **I**) of mAbs binding to egg-grown influenza viruses. Data in **D** and **H** are mean ± S.D. Data in **D** were analyzed using two-tailed Mann-Whitney tests and data in **F** were analyzed using chi-squared test. Data in **I** were analyzed using non-parametric paired Friedman tests.
