# Supplementary material for: An Egg-Derived Sulfated N-Acetyllactosamine Glycan Is an Antigenic Decoy of Influenza Virus Vaccines
Source: mBio. 2021 Jun 15;12(3):e00838-21. doi: 10.1128/mBio.00838-21 (PMC8263001; doi:10.1128/mBio.00838-21)
Supplement: TABLE S2 [file mbio.00838-21-st002.docx]

| **mAb** | **VH** | **DH** | **JH** | **VK/VL** | **JK/JL** | **H-CDR3 Length** | **HC Mutations** | **Original Isotype** | **Clone**  **(HC/LC)** |
| --- | --- | --- | --- | --- | --- | --- | --- | --- | --- |
| 029-09 3A01 | VH3-30 | DH2-21 | JH4 | VL2-8 | JL3 | 15 | 25 | IgM | 0/0 |
| 029-09 3A04 | VH3-7 | DH6-19 | JH4 | VL1-51 | JL3 | 10 | 14 | IgG | 7/19 |
| 029-09 3C04 | VH1-18 | DH6-19 | JH4 | VL1-40 | JL3 | 17 | 18 | IgM | 0/0 |
| 029-09 3C06 | VH3-7 | DH6-19 | JH4 | VL1-51 | JL3 | 10 | 14 | IgM | 7/19 |
| 029-09 3D03 | VH3-7 | DH6-6 | JH4 | VL1-51 | JL2 | 10 | 21 | IgM | 3/17 |
| 029-09 3D04 | VH3-7 | DH6-25 | JH5 | VL1-51 | JL2 | 10 | 21 | IgM | 0/17 |
| 029-09 3D06 | VH3-74 | DH3-10 | JH4 | VL2-8 | JL3 | 9 | 21 | IgM | 0/0 |
| 029-09 3F05 | VH3-7 | DH6-25 | JH4 | VL1-44 | JL3 | 10 | 6 | IgM | 2/15 |
| 029-09 3G05 | VH3-7 | DH1-26 | JH4 | VL1-51 | JL2 | 10 | 17 | IgG | 3/17 |
| 029-09 4E04 | VH3-23 | DH6-6 | JH6 | VK2-28 | JK1 | 18 | 26 | IgM | 0/0 |
| 008-10 6D02 | VH3-7 | DH6-13 | JH4 | VL1-51 | JL3 | 10 | 24 | unknown | 0/19 |
| 011-10 2A01 | VH3-7 | DH2-8 | JH5 | VL1-44 | JL3 | 11 | 5 | IgM | 0/15 |
| 011-10 2F01 | VH3-30 | DH2-21 | JH4 | VL1-51 | JL3 | 12 | 8 | IgM | 0/0 |
| 011-10 2G01 | VH3-7 | DH2-21 | JH5 | VL1-44 | JL3 | 14 | 8 | IgM | 11/15 |
| 011-10 3A03 | VH3-7 | DH2-21 | JH5 | VL1-44 | JL3 | 14 | 8 | IgM | 11/15 |
| 011-10 3B01 | VH3-7 | DH3-9 | JH4 | VL1-44 | JL3 | 7 | 18 | IgM | 0/15 |
| 011-10 3B03 | VH3-7 | None | JH4 | VL1-44 | JL3 | 7 | 4 | IgM | 9/15 |
| 017-10 3B06 | VH3-7 | DH2-15 | JH4 | VL1-44 | JL1 | 10 | 13 | IgG | 0/12 |
| 017-10 3D06 | VH3-74 | None | JH4 | VL4-69 | JL3 | 7 | 20 | IgM | 0/0 |
| 017-10 3E02 | VH3-7 | DH2-21 | JH4 | VL1-44 | JL1 | 10 | 6 | IgM | 0/12 |
| 019-10 4A06 | VH3-7 | DH4-4 | JH4 | VL1-44 | JL1 | 10 | 7 | unknown | 0/12 |
| 019-10 4E01 | VH3-7 | DH2-15 | JH4 | VL1-51 | JL1 | 7 | 9 | IgG | 0/16 |
| 034-10 4G02 | VH3-7 | DH6-13 | JH3 | VL1-44 | JL1 | 10 | 11 | IgM | 0/12 |
| 051-10 2E02 | VH3-7 | DH6-13 | JH4 | VL1-44 | JL3 | 10 | 14 | IgA | 3/15 |
| 051-10 2G03 | VH3-7 | DH1-26 | JH4 | VL1-44 | JL3 | 10 | 24 | IgG | 4/15 |
| 051-11 2F03 | VH3-7 | DH1-26 | JH4 | VL1-44 | JL3 | 10 | 22 | unknown | 4/15 |
| 051-11 5C06 | VH3-7 | DH1-26 | JH4 | VL1-44 | JL3 | 10 | 22 | unknown | 4/15 |

**Table S2: Egg-binding mAb information.** Clonal number of 0 indicates a particular chain was non-clonal.
