# Supplementary material for: An Egg-Derived Sulfated N-Acetyllactosamine Glycan Is an Antigenic Decoy of Influenza Virus Vaccines
Source: mBio. 2021 Jun 15;12(3):e00838-21. doi: 10.1128/mBio.00838-21 (PMC8263001; doi:10.1128/mBio.00838-21)
Supplement: TABLE S6 [file mbio.00838-21-st006.docx]

| **Clone** | **V** | **DH** | **J** | **CDR3 Length** | **Predicted Germline CDR3 Sequence** | **# of Members** | **Subject(s) with Clone** |
| --- | --- | --- | --- | --- | --- | --- | --- |
| **1** | H3-7 | 1-26 | H3 | 7 | ARKVGDV | 4 | 038 |
| **2** | H3-7 | 6-13 | H4 | 10 | ARAIAAAGSY | 4 | 029-09, 089 |
| **3** | H3-7 | 6-13 | H4 | 10 | ARAIAAAASR | 42 | 029-09, 051-10, 103, 038, 102, 089 |
| **4** | H3-7 | 3-10 | H4 | 10 | AREIAGRGAY | 55 | 051-10/11, 070, 089 |
| **5** | H3-7 | 6-13 | H4 | 10 | ARAIAAADSF | 12 | 103, 102, 089 |
| **6** | H3-7 | 3-22 | H4 | 10 | ARALGSGSCV | 3 | 038 |
| **7** | H3-7 | 6-19 | H4 | 10 | ARAYAGYSSY | 2 | 029-09 |
| **8** | H3-7 | 6-13 | H4 | 10 | AKSLAAADAF | 3 | 038 |
| **9** | H3-7 | None | H4 | 7 | ARRYFDY | 27 | 011-10, 038, 102, 070 |
| **10** | H3-7 | 3-10 | H5 | 10 | CARAYGSGSS | 2 | 082 |
| **11** | H3-7 | 2-21 | H5 | 14 | AGPPPGGEIAMGGS | 2 | 011-10 |
| **12** | L1-44 | N/A | L1 | 11 | AAWDDSLNGYV | 93 | 017-10, 019-10, 034-10, 120, 103, 015, 038, 070, 089 |
| **13** | L1-44 | N/A | L2 | 11 | AAWDDSLNGLV | 13 | 038, 102, 070, 108, 089, 008 |
| **14** | L1-44 | N/A | L2 | 11 | AAWDDSLNGFI | 5 | 102 |
| **15** | L1-44 | N/A | L3 | 11 | AAWDDSLNVWV | 50 | 029-09, 051-10/11, 011-10, 120, 103, 099, 030, 038, 102, 070, 108, 089, 008 |
| **16** | L1-51 | N/A | L1 | 11 | GTWDSSLSAYV | 7 | 019-10, 085, 120, 103, 070, 008 |
| **17** | L1-51 | N/A | L2 | 11 | GTWDSSLSAMV | 16 | 029-09, 103, 038, 089, 008 |
| **18** | L1-51 | N/A | L3 | 11 | GTWDSSLKSIV | 2 | 089 |
| **19** | L1-51 | N/A | L3 | 11 | GTWDSSLSAGM | 6 | 029-09, 008-10, 082, 008 |

**Table S6: Heavy and light chains clonal information.**
