# Supplementary material for: An Egg-Derived Sulfated N-Acetyllactosamine Glycan Is an Antigenic Decoy of Influenza Virus Vaccines
Source: mBio. 2021 Jun 15;12(3):e00838-21. doi: 10.1128/mBio.00838-21 (PMC8263001; doi:10.1128/mBio.00838-21)
Supplement: TABLE S7 [file mbio.00838-21-st007.docx]

| **Subject ID** | **Egg Binding mAbs (Yes/No)** | **Post Vaccination Time Point (Days)** |
| --- | --- | --- |
| 029-09 | Yes | 18 |
| 045-09 | No | 21 |
| 047-09 | No | 21 |
| 051-09 | Yes | 21 |
| 008-10 | Yes | 21 |
| 011-10 | Yes | 21 |
| 014-10 | No | 21 |
| 017-10 | Yes | 21 |
| 024-10 | No | 14 |
| 051-10 | Yes | 21 |
| 120-10 | No | 21 |

**Table S7: Serum donors for samples analyzed in Figure 1H and Figure S1D-F.**
